# Differences in Trainees’ Ageist Attitudes and Aging Anxiety by Quantity and Quality of Contact With Older Adults

**DOI:** 10.1093/geroni/igaf122.2200

**Published:** 2025-12-31

**Authors:** Grace Caskie, Maria Tice

**Affiliations:** Lehigh University, Bethlehem, Pennsylvania, United States; Lehigh University, Bethlehem, Pennsylvania, United States

## Abstract

Ageist attitudes and aging anxiety have been identified as explanations for psychology trainees’ low interest in geropsychology. Frequent, high-quality, intergenerational contact between trainees and older adults appears key to reducing ageism (Allport, 1954; Levy, 2018). However, little is known about the types or quality of contact trainees have with older adults or how quantity and quality may interact. In this study, trainees (N = 213; aged 21-35) from clinical/counseling psychology doctoral programs completed ageism, aging anxiety, and contact measures. Frequent contact occurred most often as brief (78.9%) or long (62.9%) conversations with older adults; frequent contact in other domains was less common (22.1%-54.5%). High quality contact significantly associated with more frequent contact for 10 of 11 domains. Only significant main effects of quantity and quality of contact were found for ageist attitudes and aging anxiety. Ageist attitudes were lower for those reporting frequent long talks with an older adult; aging anxiety was lower for trainees report frequent contact in five domains (long talk, older adult discussed life with them, helped older adult, older adult helped them). Ageist attitudes were lower for those with positive contact in four domains (discussed problem with older adult, helped older adult, older adult helped them, older adult coworker); aging anxiety was lower for those with positive contact in 10 of 11 domains. Encouraging intergenerational contact that occurs in one-on-one settings, involves collaboration, and includes sharing of personal information may be most likely to be associated with less ageist attitudes and aging anxiety among psychology doctoral trainees.

